# Comparative Studies of Pectinase Production by* Bacillus subtilis strain Btk 27* in Submerged and Solid-State Fermentations

**DOI:** 10.1155/2018/1514795

**Published:** 2018-12-04

**Authors:** Oliyad Jeilu Oumer, Dawit Abate

**Affiliations:** ^1^Addis Ababa University, Institute of Biotechnology, P.O. Box 1176, Ethiopia; ^2^College of Natural Science, Addis Ababa University, P.O. Box 1176, Ethiopia

## Abstract

The request for enzymes in the global market is expected to rise at a fast pace in recent years. With this regard, there has been a great increase in industrial applications of pectinase owing to their significant biotechnological uses. This study was undertaken with main objectives of meeting the growing industrial demands of pectinase, by improving the yield without increasing the cost of production. In addition, this research highlights the underestimated potential of agroresidues for the production of biotechnologically important products. In this study, the maximum pectinase production attained was using wheat bran, among the tested agroresidues. The production of pectinase was improved from 10.1 ± 1.4 U/ml to 66.3 ± 1.2 U/ml in submerged fermentation whereas it was in solid state fermentation from 800.0 ± 16.2 U/g to 1272.4 ± 25.5 U/g. The maximum pectinase production was observed using YEP (submerged fermentation) and wheat bran (solid state fermentation) at initial pH of 6.5, at 37°C and by supplementing the medium with 3 mM MgSO4.7H2O.

## 1. Background

Microbial enzymes are considered as an efficient tool for ecofriendly biotechnological progressions, as the modern society currently concentrating on green biotechnology. Pectinases are a group of enzymes that contribute to the degradation of pectin, which is a complex acidic polysaccharide present in the primary cell wall and middle lamella of higher plant tissues. The significance of these enzymes for the development of environment friendly industrial processes has already been established [1].

In various industrial sectors whenever pectin degradation is needed pectinolytic enzymes can be applied. Numerous microorganisms have been known and used to produce different types of pectinolytic enzymes [2]. About 25% of the global food and industrial enzyme sales accounts by microbial pectinases [3, 4] and their market is increasing day by day. The applications of pectinase include fruit juice clarification, juice extraction, refinement of vegetable fibers, degumming of natural fibers, and wastewater treatment and act as an analytical tool in the assessment of plant products [4–6]. Pectinase usage accelerates tea fermentation and also destroys the foam forming property of instant tea powders by destroying pectins. They are also used in coffee fermentation to remove mucilaginous coat from coffee beans [7–9].

As many other enzyme production techniques, there are two fermentation methods that we can use for pectinases production, which are solid state fermentation (SSF) and submerged fermentation (SmF). Solid state fermentation is well defined as the cultivation of microorganisms on moist solid supports with very little amount of moisture content/water. In contrast, in submerged fermentation (SmF) the nutrients and microorganisms are both submerged in water (Singh Nee Nigam and Pandey 2009).

It is estimated that about 90% of all industrial enzymes are produced in submerged fermentation because SmF is much easier for accessing and scaling up the production process. In this respect SmF processing offers an insurmountable advantage over SSF. However, solid state fermentations have numerous rewards over submerged fermentations including higher concentration of products and less effluent generation [10].

Higher cost of the production is perhaps the major constraint in commercialization of new sources of enzymes. Though, using high yielding strains, optimal fermentation conditions and cheap raw materials as a carbon source can reduce the cost of enzyme production for subsequent applications in industrial processes [4]. In this view, agroindustrial waste materials can be used both as source of energy for growth and as carbon for synthesis of cell biomass and other products. With this regard, solid state fermentation (SSF) permits the use of agricultural and agroindustrial residues as substrates which are converted into bulk chemicals and fine products with high commercial value. The selection of a substrate for enzyme production in an SSF process depends on several factors, mainly related with cost and availability of the substrate ([1]; Singh Nee Nigam and Pandey 2009). As agroindustrial residues are renewable and in an abundant supply (~3.5 billion tonnes/year), they represent a potential low cost raw material for microbial enzyme production (Singh Nee Nigam and Pandey 2009).

Previously, we endeavored to screen microorganism for pectinase production and examine their potential in mucilage removal from coffee beans [9]. Herein study, we attempt to advance the economical and ecofriendly productivity of pectinase from* Bacillus subtilis strain *Btk 27. In addition, we attempted a very diligent and comprehensive SmF and SSF comparative pectinase production optimizations.

## 2. Material and Methods

### 2.1. Inoculum Preparation

Fresh culture of* Bacillus subtilis strain *Btk 27 was inoculated into sterilized yeast extract pectin (YEP) medium. The pH of the medium was adjusted at 7.0 ± 0.5. The inoculated flask was incubated at 30°C on a rotary shaker at 120 rpm. Culture was grown in 50 ml media in 250 ml Erlenmeyer flasks. This inoculum was used for subsequent experiments.

### 2.2. Pectinase Enzyme Assay

Pectinase enzyme assay was based on the determination of reducing sugars produced as a result of enzymatic hydrolysis of pectin by dinitro salicylic acid reagent (DNS) method (Miller, 1959). For enzyme assay, 1.5 mL of freshly grown culture was taken and centrifuged at 10,000 rpm for 5 min. The supernatant (100 *μ*L) from the culture broth was served as the source of the enzyme. The enzyme unit was defined as the amount of enzyme that catalyzes *μ*mol of galacturonic acid per minute (*μ*mol min^−1^) under the assay conditions. Relative activity was calculated as the percentage enzyme activity of the sample with respect to the sample for which maximum activity is obtained.

(1) $$\begin{array}{c} \text{Relative Activity}=\frac{Activity\;\;of\;\;sample\;\;\left(U\right)\times 100}{Maximum\;\;enzyme\;\;activity\;\;\left(U\right)} \end{array}$$

### 2.3. Effect of Nutrient Media

The effect of nutrient media on the production of pectinase in submerged fermentation was studied using Yeast extract, Luria-Bertani broth, Nutrient broth, Peptone, Trypton soybean meal, and Malt extract. Each nutrient media (1% w/v) was supplemented with 0.25% (w/v) Apple pectin. The pH of the nutrient media was adjusted to 7.0 ± 0.5 and sterilized. 50 mL of nutrient media in 250 mL Erlenmeyer flasks were inoculated with 1% (v/v) of inoculum and incubated at 30°C, 120 rpm for 48 hours. After incubation, samples were collected and centrifuged at 10,000 rpm for 5 min at 4°C. The supernatant was used for measuring the enzyme activity. The Pectinase activity was determined in the supernatant as U/ml.

### 2.4. Effect of Agro Residues (Substrate)

Agricultural residues such as Coffee pulp, Orange peel, and lemon peel and wheat bran were used as substrate for solid state fermentation. In 250 ml conical flask, 5.0 g of each agro residue was moistened by 60% of distilled water and autoclaved at 121°C for 15 minutes. The flasks were inoculated with 2.0 ml of inoculum, mixed well to evenly distribute the inoculum and incubated at 37°C for 48 h.

### 2.5. Extraction of Pectinase from the Solid Substrate

Extraction of Pectinase from SSF was done according to the method of Xiros* et al*. 2008. After 48 hour of incubation 50 ml of distilled water was added into the solid substrate, shaken the flasks for 1 h at 120 rpm on orbital shaker thoroughly and slurry is formed. Then, the flasks were kept at 4°C for 30 min under static conditions to facilitate the enzyme extraction. The slurry was centrifuged at 10,000*g *for 10 min at 4°C, and the clear supernatant was collected to assay the pectinase activity. The Pectinase activity was determined in the supernatant as U/g of solid substrate used.

### 2.6. The Effect of Moisture Content

To study the effect of moisture content on the production of pectinase enzyme using SSF, the optimized solid substrate was moistened at 45%, 55%, 65%, 75% and 85% moisture content using distilled water before sterilization. Then, the autoclaved substrate was inoculated with 2 ml of inoculum and incubated at 37°C for 48 h. After the end of incubation, the pectinase activity was determined.

### 2.7. Effect of pH

The pH of the optimized nutrient media and agroresidue was adjusted to pH that ranges from 4.0-9.0 with 0.5 intervals before sterilization. The sterilized nutrient medium and solid substrate were inoculated and incubated at 37°C, 120 rpm (for SmF only), for 48 hours.

### 2.8. Effect of Temperature

The sterilized and optimized agroresidue and nutrient media were inoculated and incubated at 25°C, 30°C, 37°C, 40°C, 45°C and 50°C for 48 h to study the effect of temperature on enzyme production.

### 2.9. Effect of Agitation

To study the effect of agitation on SmF, the optimized nutrient media was inoculated and incubated at different speeds such as static (0), 120 rpm, 150 rpm and 180 rpm at optimized temperature.

### 2.10. Effect of Inoculum Size

To examine the effect of inoculums size on pectinase production, the optimized nutrient media and agroresidue were inoculated with various inoculum sizes such as 0.5% v/v, 1% v/v, 2% v/v, 3% v/v and 4% v/v for SmF and 5% v/v, 10% v/v, 15% v/v, and 20% v/v for SSF.

### 2.11. Effect of Salts

To study the effect of salts on pectinase production, the optimized nutrient media and agroresidue were supplemented with 3 mM of various salts such as: CaCl_2_.2H_2_O, MgSO_4_.7H_2_O, CuCl_2_.2H_2_O, CoCl_2_.2H_2_O, ZnCl2, FeSO_4_.7H_2_O, and NaCl. In SSF, these salts were dissolved in the distilled water which was used for adjusting moisture level before incorporating them into the solid substrate.

### 2.12. Effect of Carbon Sources

To examine the effect of carbon sources on Pectinase production both in SmF and SSF, various carbon sources such as dextrose, fructose, arhabinose, galacturonic acid, galactose, sucrose, and xylose were supplemented into optimized nutrient media and agro residues at a concentration of 1% w/v along with 0.25% Apple pectin (in case of SmF). In case of SSF, these carbon sources were dissolved in the distilled water which was used for adjusting moisture level before incorporating them into the solid substrate.

### 2.13. Effect of Nitrogen Sources

The effect of Nitrogen sources on pectinase production both in SmF and SSF were studied by supplementing various organic and inorganic nitrogen sources, namely casein, peptone, tryptone, glycine, urea, ammonium chloride, ammonium nitrate, ammonium sulfate of 1% (w/v) into optimized nutrient media and agro residue. In case of SSF, these nitrogen sources were dissolved in the distilled water which was used for adjusting moisture level before incorporating them into the solid substrate.

### 2.14. Effect of Vitamins

To examine the effect of vitamin on pectinase production both in SmF and SSF, the optimized medium and agroresidue were sterilized and supplemented with different concentrations of multivitamin solution such as 0.1% v/v, 0.2% v/v, 0.3% v/v, and 0.4% v/v.

### 2.15. Effect of Time of Incubation

To study the effect of incubation time, within 12-hour interval aliquots of samples were taken and the pectinase activity was assayed.

### 2.16. Data Analysis

All statistical analyses were performed using experimental results which were expressed as means ± SD of three parallel replicates. Mean of the results were compared using post- hoc multiple comparison analysis performed using Tukey homogenous test using GraphPad Prism 5 software at a significance level of p< 0.05. The results were analyzed using Origin pro 8 data analysis and GraphPad Prism 5 desktop version software.

## 3. Results

### 3.1. Nutrient Media

The inoculated nutrient media were incubated at 30°, 120 rpm for 48 hours and assayed for pectinase activity at the end of incubation. The highest pectinase production attained was using yeast extract (10.1 ± 1.4 U/ml). Furthermore; the production of pectinase in yeast extract was significantly higher than any of the other media (Table 1).

### 3.2. Effect of Agro Residues

Among the studied agroresidues, the maximum pectinase activity achieved was 800.0 ± 16.2 U/g using wheat bran. In contrast, the lowest pectinase production was 93.4 ± 7.3 U/g from coffee husk (Table 2). Production of pectinase using coffee pulp, lemon peel and orange peel were not significantly different. Therefore, the subsequent SSF studies were carried out using wheat bran as substrate.

### 3.3. Moisture Content

**Figure 1:**
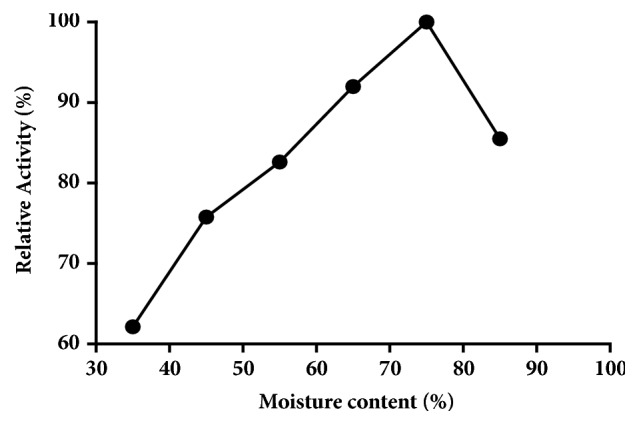
Effect of moisture content on pectinase production.

In order to study the effect of moisture content on SSF, wheat bran was moistened at a range of 35 - 85% moisture content using distilled water. The maximum pectinase production was at 75% initial moisture content (Figure 1).

### 3.4. Effect of pH

**Figure 2:**
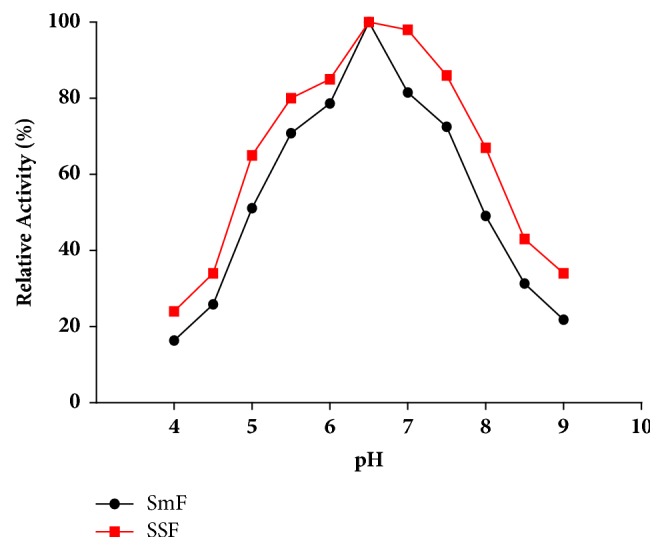
Effect of initial pH of growth media on pectinase production.

To study the effect of initial pH of growth media both on SmF and SSF pectinase production, YEP and wheat bran were adjusted to a pH range of 4.0 - 9.0. In both fermentations, the maximum pectinase activity attained was at the 6.5 initial pH (Figure 2).

### 3.5. Effect of Temperature

The wheat bran with appropriate moisture and as well YEP, were inoculated and incubated at various temperatures. The maximum pectinase production attained was at 37°C for both fermentation techniques (Table 3). Thus, the succeeding studies were performed at incubation temperature of 37°C.

### 3.6. Effect of Agitation

To study the effect of agitation on submerged fermentation, the inoculated YEP was incubated at optimized conditions with different agitation speed. An enzyme activity of 13.1 ± 1.8 U/ml was recorded at 120 rpm which was the highest. In contrast, 7.2 ± 0.4 U/ml was found to be the lowest at the agitation speed of 0 rpm (Table 4).

### 3.7. Effect of Inoculum Size

The effect of inoculum size on both fermentation techniques was studied and the highest pectinase production in SmF achieved was 15.4 ± 0.4 U/ml at 1% v/v inoculum size (Table 5). The subsequent SmF studies were performed at 1% v/v inoculum size. Whereas in case of SSF, the maximum pectinase production achieved was 1018.1 ± 47.8 U/g using 10% v/v inoculums size (Table 5).

### 3.8. Effect of Salts on Pectinase Production

YEP and Wheat bran were supplemented with 3.0 mM of different salts to study their effects on productivity of pectinase. In case of SmF, CaCl_2_.2H_2_0, MgSO_4_.7H_2_O, CoCl_2_.6H_2_0 and NaCl significantly enhanced the enzyme production compared to the control. Both CaCl_2_.2H_2_0 and MgSO_4_.7H_2_O significantly increased pectinase activity by three folds. The maximum pectinase production attained was 54.0 ± 2.5 U/ml by supplementing YEP with MgSO_4_.7H_2_O (Table 6).

In case of SSF supplementation of wheat bran with CaCl_2_.2H_2_0, MgSO_4_.7H_2_O and NaCl showed enhanced trend of pectinase production although not significant. The maximum pectinase production observed was 1169.7 ± 147.8 U/g by supplementing MgSO_4_.7H_2_O (Table 6). However, FeSO_4_.7H_2_0 and ZnSO_4_.7H_2_O significantly reduced pectinase production. The lowest pectinase activity achieved was 566.9 ± 51.0 U/g by ZnSO_4_.7H_2_O. The subsequent SmF and SSF studies were performed by supplementing 3 mM of MgSO_4_.7H_2_O into YEP and wheat bran.

### 3.9. Effect of Carbon Sources

YEP and wheat bran were supplemented with 1% of different carbon sources along with 3.0 mM of MgSO_4_.7H_2_O to study their effect. In case of SmF, supplementation with carbon sources (except for sucrose) significantly decreased the pectinase production (Table 7). The highest pectinase production achieved was on the control. Therefore, the subsequent SmF studies were carried out on YEP in the presence of 0.25% apple pectin without any other carbon source.

In case of SSF, the highest activity attained was 1172.3 ± 24.68 U/g in the control which was not supplemented by any carbon source (Table 7). Therefore, the subsequent SSF studies were also carried out without any carbon source supplementation.

### 3.10. Effect of Nitrogen Sources

To study the effect of nitrogen sources on pectinase production, YEP and wheat bran were supplemented with different nitrogen sources at 1% (w/v). In case of SmF, the highest pectinase production was at 67.7 ± 4.7 U/ml by supplementing Yeast extract with casein. The other tested nitrogen sources significantly decreased pectinase production. Where as in case of SSF, the highest pectinase production observed was 1261.2 ± 64.0 U/g when supplemented with ammonium sulphate (NH_4_SO_4_). However, the effect wasn't significant (Table 8).

### 3.11. Effect of Vitamins

To study the effect of vitamins on pectinase production, multivitamin solution was incorporated into YEP and Wheat bran. There was no significant effect on SmF pectinase production, however, significant declining of enzyme production was observed on SSF (Table 9).

### 3.12. Effect of Incubation Period

**Figure 3:**
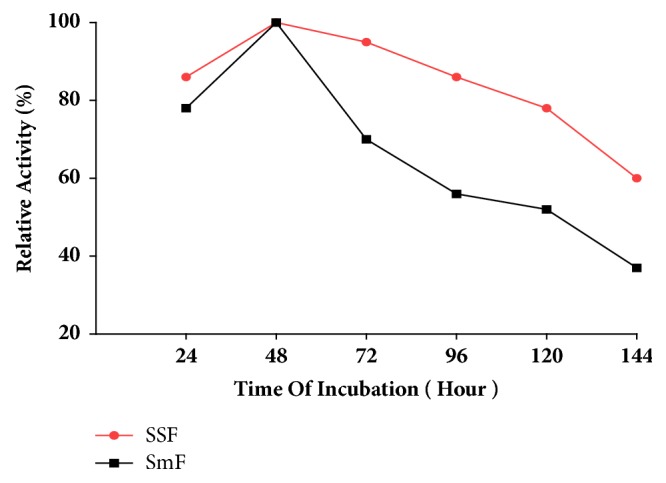
Effect of Time of Incubation on Pectinase production.

To study the effect of incubation period, the inoculated YEP and Wheat bran were assayed for pectinase activity within 24 hours interval. Accordingly, the highest pectinase enzyme production on both SmF and SSF was achieved at 48 hours of incubation. Beyond 48 hour of incubation the production of pectinase both on SmF and SSF, declined (Figure 3).

## 4. Discussions

Emerging new applications of pectinase, underline the importance of screening pectinase producing microorganisms with novel properties, greater enzyme activity and large-scale production of these enzymes [11]. The potential of microorganisms to produce extracellular enzymes is influenced by environmental conditions such as temperature, pH, aeration, inoculums and the presence of inducer or repressor substrates [12]. In this study, parameters that affect the pectinase production have been standardized and diligent optimization steps were carried out to make the production of pectinase enzyme to be cost effective and commercially viable. Since, to meet the growing industrial demands for pectinase, it is necessary to improve yield without increasing the cost of production. Thus, in this study the biotechnological capacities of agricultural wastes are considered for economical production of pectinase. In addition, a comprehensive comparative SmF and SSF optimization studies are undertaken.

In this study, among the tested nutrient media, the highest production of Pectinase on submerged fermentation was 10.1 ± 1.4 U/ml using Yeast Extract. The result is in agreement with; Kashyap et al., [13] reported the combination of Yeast Extract with pectin to be the best medium for pectinase production.* Bacillus shaericus *MTCC 7542 produced maximum polygalactouronase when grown on mineral medium containing yeast extract as sole nitrogen source [2]. Yeast extract is the best nitrogen source for pectinase production, probably due to its high content in minerals, vitamins, coenzymes and nitrogen components.

Among the tested agroresidues for pectinase production, maximum enzyme production on solid state fermentation achieved was 800.0 ± 16.2 U/g from wheat bran. In the same way, Namasivayam et al. 2011 working on* B. cereus *isolated from market solid waste reported that pectinase production was enhanced by wheat bran. Of the various substrates reported in the literature, wheat bran has been the prime among all [10]. El-Shishtawy et al. 2014 conducted solid state production of pectinase from B. megatherium using wheat bran, grasses, palm leaves, and date seeds and the maximum pectinase achieved was 350 U/g using wheat bran. Wheat bran characterized by its better air circulation, loose particle binding and efficient penetration, and cheaper; therefore it showed a better prospect economically in fermentation processes [14].

Moisture is one of the most important parameter in solid state fermentation (SSF) that influences the growth of the organism and thereby enzyme production. Moisture is reported to cause swelling of the substrates, thereby facilitating better utilization of the substrate by microorganisms [14]. The maximum pectinase production from* Bacillus subtilis *strain Btk27 was recorded at 75% initial moisture content. Kashyap et al. 2003 also reported that 75% initial moisture content for enhanced production of pectinase by* Bacillus sp*. DT7. The moisture level in SSF process varies between 70 and 80% for bacteria [10]. Any further increase in moisture content resulted in the decrease of enzyme yields may be due to clumping of solid particles which results in the decrease of interparticle space and diffusion of nutrients. In contrast, the low moisture content leads to the decreased solubility of nutrients present in the wheat bran thereby decreased enzyme yields.

The initial pH of the fermentation medium plays a vital role in determining the level of metabolite synthesis. The stability of the microbial metabolite is also dependent on the hydrogen ion concentration of the medium. In present study the maximum pectinase production attained both on solid state fermentation and on submerged fermentation was at the 6.5 initial pH. It has been reported that optimum pH in both cases of fermentation SSF and SmF was similar. This may be due to the fact that the optimum pH for the production of pectinase is more related to the optimum conditions required for the growth of specific microorganism employed to conduct the fermentation than other factors, so it may have remained in a particular range for some microorganism, irrespective of the type of fermentation [15]. These results are in agreement with the following: Banu et al. [16] also found that* P. chrysogenum* exhibited maximum polygalacturonase production at initial pH of 6.5. The pectinase produced by* Bacillus sphaericus *(MTCC 7542) had the maximum activity at pH 6.8 initial pH of Medium [2].

Temperature is very important factor for microbial growth as well as microbial product formation. The incubation temperature greatly affects the microbial growth rate, enzyme secretion, enzyme inhibition, and protein denaturation [11]. In this study the maximum pectinase production was observed at 37°C for both on submerged and solid-state fermentations. The result is in good agreement with; Namasivayam et al. [17] reported an optimum temperature for maximum activity of pectinase from* B. cereus *to be 37°C. The optimum temperature for pectinase production was found to be 37°C whereas no other temperature was suitable to such extent for growth and enzyme secretion [18].

Agitation plays a vital role in mass transfer in a submerged fermentation. In this study agitation increased pectinase production significantly. Kashyap et al. [13] reported that aeration has a significant influence on the pectinase production by* Bacillus sp*. DT7. Darah et al. [19] explained that, at lower agitation speed, the inadequate mixing of the broth towards the later stages of growth affected the enzyme synthesis, while the drastic dropping in enzyme activity at higher agitation speeds was due to shearing effect on the cells.

The initial load of microorganisms also influences the final level of the enzyme synthesized. In this study, the maximum enzyme production observed was at 1% v/v inoculum size and at 10% v/v in case of SmF and SSF, respectively. The results are in agreement with the following: Ahlawat et al. [18] reported SmF pectinase production by* Bacillus subtilis *at inoculums size of 1% (v/v) was much higher compared to 2% (v/v). Kashyap et al. [20] reported that 10% (w/v) of an inoculum size for SSF production of pectinase using* Bacillus *sp. DT7. Adequate nutrient supply could be the reason of the higher enzyme production with optimum inoculums size. Also, the pectinase production reduction beyond optimum inoculums size could be due to rapid depletion of nutrients and development of oxygen stress resulting from a high microbial load.

In this study, CaCl_2_.2H_2_0, CoCl_2_.6H_2_0, MgSO_4_.7H_2_O, and NaCl enhanced pectinase production on submerged fermentation. Both CaCl_2_.2H_2_0 and MgSO_4_.7H_2_O significantly increased pectinase production by three folds. These results are in agreement with the following: Kashyap et al. [13] reported more than three-fold increase in pectinase production by supplementing MgSO_4_ and CaCl_2_. While CaCl_2_.2H_2_0, MgSO_4_.7H_2_O, and NaCl also increased pectinase production on solid state fermentation however their effect was not significant. The maximum pectinase activity observed was 1169.7 ± 147.8 U/g by supplementing MgSO_4_.7H_2_O. Banu et al. [16] observed little effect of Mg^2+^ and Ca^2+^ on pectinase from* P. chrysogenum*.

An adequate supply of carbon as energy source is critical for optimum growth affecting the growth of organism and its metabolism. In the present study, the maximum pectinase production observed both on submerged fermentation and solid-state fermentation were on the controls. Supplementing carbon sources decreased pectinase production on both solid state and submerged fermentations. According to Ahlawat et al., [18] low enzyme production with other carbon sources is might be because of catabolite repression. Glucose is known to repress the transcription of genes encoding enzymes required for the utilization of alternative carbon sources; some of these genes are also repressed by other sugars such as galactose, sucrose, and arabinose and the process is known as catabolite repression [21, 22]. This result agrees with the study of Solís -Pereira et al., [23] where the production of polygalacturonase was lower when free sugars were added to the medium compared to the presence of pectin as the sole carbon source in submerged fermentation. Fawole and Odunfa [24] found that pectin and polygalacturonic acid promoted the production of pectic enzyme. Phutela et al. [25] stated that pure pectin and wheat bran supported maximum pectinase production. The same carbon supplements except starch caused repressive effect on pectinase production by* B. licchenformis* [26].

Nitrogenous compounds are utilized by the microbial cells for the synthesis of nucleotides, amino acids, proteins, enzymes, and other metabolites [27]. Nitrogen supplements, when incorporated into the production medium, facilitate better biomass production and subsequently higher metabolite secretion. In this study, the maximum pectinase production attained on submerged fermentation was 67.7 ± 4.7 U/ml by supplementing Casein. Similar results have been reported by other workers; Thakur et al. [28] reported that combination of casein hydrolysate and yeast extract gave high yield of polygalacturonase from* Mucor circinelloides *ITCC 6025. Jayani et al., [2] working on* Bacillus sphaericus* (MTCC 7542), reported that a combination of yeast extract and casein hydrolysate also gave high polygalacturonase activity. Of the various nitrogen sources used, maximum pectinase activity was observed when casein hydrolysate and yeast extract were used together [2]. Meanwhile, among the tested nitrogen sources, ammonium sulphate (NH_4_SO_4_) and ammonium nitrate (NH_4_NO_3_) increased the pectinase productivity on SSF though their effect was not significant. The result is in good agreement with Fawole and Odunfa [24] who found that ammonium sulphate and ammonium nitrate were good nitrogen sources for pectic enzyme production from* A. niger*. Moreover, Sarvamangala and Dayanand [29] revealed that ammonium sulphate did influence production of pectinase positively in solid-state conditions.

In present study, there wasn't any significant effect of pectinase production on submerged fermentation by supplementation of multivitamin. However, supplementing vitamin significantly decreased SSF pectinase production. According to Kashyap et al., [20], Pectinase production was enhanced by 65.8% when multivitamin solution was added to wheat bran. Similarly, Kashyap et al. [13] reported that supplementing multivitamin solution increased* Bacillus sp.* DT7 pectinase production by 61% on submerged fermentation. However, the results of this study are in contrast with the above reports. This could be due to the multivitamin solution in this study contained ZnSO_4_.7H_2_O as a component. As it observed in this study, by supplementing ZnSO_4_.7H_2_O there was no significant effect on pectinase production using submerged fermentation; however, it significantly decreased pectinase production on solid state fermentation.

The time of fermentation had a profound effect on microbial product formation [4]. The level of enzyme production varies with the time duration of the fermentation process. In this study, the pectinase activity was increased continuously until 48 hours of incubation. Onwards 48 hour of incubation the pectinase activity was decreased. Thus, optimum time of pectinase synthesis was to be 48 hour after inoculation [30]. The reduction in pectinase production after 48 h might be the result of change in pH during fermentation, denaturation, or decomposition of enzyme due to interaction with other components of medium and depletion of nutrients in the medium [31].

In conclusion, Kashyap 2000 has reported that after optimizing growth conditions the pectinase production using submerged fermentation from* Bacillus* sp DT7 was 53 U/ml, which was the highest report in the literature. Nevertheless, in our study we report that a pectinase activity of 69.6 u/ml from optimized submerged fermentation. In addition, El-Shishtawy, 2014, has stated that solid state production of pectinase enhanced from 350 U/g to 610 U/g. Herein, the pectinase production from* Bacillus subtilis* strain Btk 27 in solid state fermentation improved from 800 U/g to 1272 U/g.

## 5. Conclusion

In this study, a very assiduous and all-embracing optimization steps are carried out. The production of pectinase was enhanced more than a 6-fold in submerged fermentation and a fold in solid state fermentation. The potential of agricultural wastes for the production of pectinase using solid state fermentation is highlighted in this study. In addition, for the highest productivity of pectinase from* Bacillus subtilis* strain Btk 27 both on submerged and solid-state fermentations, only adjustment of the inoculum size and temperature without supplementing carbon source, nitrogen source, and vitamin is adequate. This result conveys the very economized production of pectinase.

## Figures and Tables

**Table 1:** Effect of nutrient media on pectinase production.

**Nutrient media**	**Enzyme activity (U/ml) ** **∗**
Yeast extract	10.1 ± 1.4^a^
Luria-Bertani	6.0 ± 0.8^c^
Peptone	6.3 ± 0.2^b^
Tryptone soybean meal	5.0 ± 1.4^c^
Malt extract	4.8 ± 0.1^c^
Nutrient broth	3.5 ± 0.1^c^

(i) *∗*Values are mean ± S.D. of 3 replicates.

(ii) Values followed by different superscripts are significantly different at (*P<*0.05).

(iii) Values followed by same superscripts are not significantly different at (*P<*0.05).

**Table 2:** Effect of agroresidues on Pectinase production.

**Agro Residues**	**Enzyme activity (U/g) ** **∗**
Coffee	93.4 ± 7.3^a^
Lemon	136.8 ± 51.1^a^
Orange	113.6 ± 2.4^a^
Wheat bran	800.0 ± 16.2^b^

(i) *∗*Values are mean ± S.D. of 3 replicates.

(ii) Values followed by different superscripts are significantly different at (*P<*0.05).

(iii) Values followed by same superscripts are not significantly different at (*P<*0.05).

**Table 3:** Effect of Incubation temperature on pectinase production.

**Incubation temperature (**°**C)**	SmF	SSF
	**Relative activity (**%**)**	**Relative activity (**%**)**
**25**	56.3^a^	50.3^a^
**30**	76.7^b^	74.2^b^
**37**	100.0^c^	100.0^c^
**40**	84.0^b^	86.0^b^
**45**	42.0^d^	52.1^a^
**50**	0.1^e^	8.3^d^

(i) Values followed by different superscripts are significantly different at (*P<*0.05).

(ii) Values followed by same superscripts are not significantly different at (*P<*0.05).

**Table 4:** Effect of agitation speed on pectinase production.

**Agitation (rpm)**	**Enzyme Units (U/ml) ** **∗**	**Relative Activity (**%**)**
**0**	7.2 ± 0.4^a^	55.0
**120**	13.1 ± 1.8^b^	100.0
**150**	11.7 ± 1.2^b^	89.3
**180**	11.1 ± 0.6^b^	84.7

(i) *∗*Values are mean ± S.D. of 3 replicates.

(ii) Values followed by different superscripts are significantly different at (*P<*0.05).

(iii) Values followed by same superscripts are not significantly different at (*P<*0.05).

**Table 5:** Effect of inoculum size on pectinase production.

**Inoculum Size (**%**)**	**SmF**		**Inoculum Size (**%**)**	**SSF**	
	**Enzyme activity (U/ml) ** **∗**	**Relative activity (**%**)**		**Enzyme activity (U/g) ** **∗**	**Relative activity (**%**)**
**0.5**	13.9 ± 1.0^a^	90.3	**5**	817.4 ± 6^a^	80.3
**1**	15.4 ± 0.4^a^	100.0	**10**	1018.1 ± 47.8^b^	100.0
**2**	14.2 ± 0.2^a^	92.2	**15**	841.8 ± 60.4^a^	82.7
**3**	10.2 ± 1.4^b^	66.2	**20**	735.8 ± 48.0^a^	72.3

(i) *∗*Values are mean ± S.D. of 3 replicates.

(ii) Values followed by different superscripts are significantly different at (*P<*0.05).

(iii) Values followed by same superscripts are not significantly different at (*P<*0.05).

**Table 6:** Effect of salts on pectinase production.

**Metal Ions**	**SmF**		**SSF**	
	**Enzyme activity**	**Relative Activity**	**Enzyme activity**	**Relative Activity**
	**(U/ml) ** **∗**	**(**%**)**	**(U/g) ** **∗**	**(**%**)**
**CaCl**_**2**_ **.2H**_**2**_ **0**	48.8 ± 4.2^a^	302.5	1159.1 ± 100.1^a^	115.3
**CoCl**_**2**_ **.6H**_**2**_ **0**	40.4 ± 3.6^b^	252.5	974.0 ± 41.7^ab^	96.9
**FeSO**_**4**_ **.7H**_**2**_ **0**	8.8 ± 1.3^c^	55.0	766.1 ± 91.2^b^	76.2
**MgSO**_**4**_ **.7H2O**	54.0 ± 2.5^a^	337.5	1169.7 ± 147.8^a^	116.3
**NaCl**	44.6 ± 5.1^ab^	278.8	1025.6 ± 135.1^a^	102.3
**ZnSO**_**4**_ **.7H**_**2**_ **O**	14.3 ± 0.4^4c^	89.4	566.9 ± 51.0^b^	56.4
**Control**	16.0 ± 1.4^c^	100.0	1005.4 ± 47.8^a^	100.0

(i) *∗*Values are mean ± S.D. of 3 replicates.

(ii) Values followed by different superscripts are significantly different at (*P<*0.05).

(iii) Values followed by same superscripts are not significantly different at (*P<*0.05).

(iv) The control is not supplemented with salts.

**Table 7:** Effect of carbon sources on pectinase production.

**Carbon sources**	**SmF**		**SSF**	
	**Enzyme activity**	**Relative Activity**	**Enzyme activity**	**Relative Activity (**%**)**
	**(U/ml) ** **∗**	**(**%**)**	**(U/g) ** **∗**	
**Arhabinose**	8.5 ± 2.1^a^	14.7	769.2 ± 99.54^a^	65.6
**Dextrose**	43.4 ± 2.9^b^	75.2	1045.7 ± 316.2^a^	89.2
**Fructose**	42.1 ± 0.2^b^	73.0	946.1 ± 186.0^a^	80.7
**Galactose**	14.1 ± 5.3^ac^	24.4	1045.7 ± 232.2^a^	89.2
**D-Galacturonic Acid**	20.9 ± 7.0^c^	36.2	666.5 ± 280.7^a^	56.9
**Pectin**	-	-	836.5 ± 100.3^a^	71.4
**Sucrose**	52.0 ± 3.4^d^	90.1	781.9 ± 233.3^a^	66.7
**Xylose**	13.6 ± 1.6a^c^	23.6	707.2 ± 62.17^a^	60.3
**Control**	57.7 ± 6.04^d^	100.0	1172.3 ± 24.68^a^	100.0

(i) *∗*Values are mean ± S.D. of 3 replicates.

(ii) Values followed by different superscripts are significantly different at (*P<*0.05).

(iii) Values followed by same superscripts are not significantly different at (*P<*0.05).

(iv) The control is unsupplemented with any carbon source.

**Table 8:** Effect of nitrogen sources on pectinase production.

**Nitrogen Sources**	**SmF**		**SSF**	
	**Enzyme activity**	**Relative Activity (**%**)**	**Enzyme activity**	**Relative Activity**
	**(U/ml) ** **∗**		**(U/g) ** **∗**	**(**%**)**
**NH**_**4**_ **Cl**	44.7 ± 4.8^a^	73.4	975.7 ± 184.0^a^	84.1
**NH**_**4**_ **NO**_**3**_	29.4 ± 1.6^b^	48.3	1230.0 ± 30.2^a^	106.0
**NH**_**4**_ **SO**_**4**_	34.9 ± 2.4^b^	57.3	1261.2 ± 64.0^a^	108.7
**Casein**	67.7 ± 4.7^c^	111.2	1008.6 ± 19^a^	87.0
**Glycine**	33.9 ± 0.7^b^	55.7	970.5 ± 43.0^ab^	83.7
**Peptone**	33.8 ± 2.4^b^	55.5	933.4 ± 31.8^ab^	80.5
**Urea**	32.2 ± 3.7^b^	53.0	884.7 ± 83.4^b^	76.3
**Yeast Extract**	-	-	1076.9 ± 17.2^a^	92.8
**Control**	60.9 ± 1.1^c^	100.0	1160.0 ± 2.50^a^	100.0

(i) *∗*Values are mean ± S.D. of 3 replicates.

(ii) Values followed by different superscripts are significantly different at (*P<*0.05).

(iii) Values followed by same superscripts are not significantly different at (*P<*0.05).

(iv) The control is unsupplemented with any nitrogen source.

**Table 9:** Effect of vitamins on pectinase production.

**Vitamin (**µ**l)**	**SmF**		**SSF**	
	**Enzyme activity**	**Relative Activity (**%**)**	**Enzyme activity**	**Relative Activity (**%**)**
	**(U/ml) ** **∗**		**(U/g) ** **∗**	
**50**	65.2 ± 3.6^a^	98.3	858.6 ± 50.9^a^	67.5
**100**	68.6 ± 7.5^a^	103.5	746.0 ± 80.3^a^	58.6
**150**	64.8 ± 5.8^a^	97.7	814.9 ± 16.3^a^	64.0
**200**	69.6 ± 6.5^a^	104.9	787.8 ± 48.9^a^	61.9
**Control**	66.3 ± 1.2^a^	100.0	1272.4 ± 25.5^b^	100.0

(i) *∗*Values are mean ± SD of 3 replicates.

(ii) Values followed by different superscripts are significantly different at (*P<*0.05.

(iii) Values followed by same superscripts are not significantly different at (*P<*0.05).

(iv) The control is unsupplemented with vitamin.

## Data Availability

The data used to support the findings of this study are included within the article.

## References

[1] Jacob N. (2009). Pectinolytic enzymes. *Biotechnology for Agro-Industrial Residues Utilisation: Utilisation of Agro-Residues*.

[2] Jayani R. S., Shukla S. K., Gupta R. (2010). Screening of bacterial strains for polygalacturonase activity: its production by bacillus sphaericus (MTCC 7542). *Enzyme Research*.

[3] Jayani R. S., Saxena S., Gupta R. (2005). Microbial pectinolytic enzymes: a review. *Process Biochemistry*.

[4] Murad H. A., Azzaz H. H. (2011). Microbial pectinases and ruminant nutrition. *Research Journal of Microbiology*.

[5] Arunachalam C., Asha S. (2010). Pectinolytic enzyme—a review of new studies. *Biotechnology Advances*.

[6] Khan M., Nakkeeran E., Umesh-Kumar S. (2013). Potential application of pectinase in developing functional foods. *Annual Review of Food Science and Technology*.

[7] Hoondal G., Tiwari R., Tewari R., Dahiya N., Beg Q. (2002). Microbial alkaline pectinases and their industrial applications: A review. *Applied Microbiology and Biotechnology*.

[8] Sieiro C., García-Fraga B., López-Seijas J., Da Silva A. F., Villa T. G. Microbial Pectic Enzymes in the Food and Wine Industry.

[9] Oumer O. J., Abate D. (2017). Characterization of Pectinase from Bacillus Subtilis Strain Btk 27 and Its Potential Application in Removal of Mucilage from Coffee Beans. *Enzyme Research*.

[10] Pandey A., Soccol C. R., Nigam P., Soccol V. T., Vandenberghe L. P. S., Mohan R. (2000). Biotechnological potential of agro-industrial residues. II: cassava bagasse. *Bioresource Technology*.

[11] Singh S., Mandal S. K. (2012). Optimization of processing parameters for production of pectinolytic enzymes from fermented pineapple residue of mixed aspergillus species. *Jordan Journal of Biological Sciences*.

[12] El-Refai A., M Metwalli S., A El-Sebaiy L. (1984). Influence of PH, inoculum, aeration and growth period on production of pectinolytic enzymes by penicillium awamori 16. *Chemie, Mikrobiologie, Technologie der Lebensmittel*.

[13] Kashyap D. R., Chandra S., Kaul A., Tewari R. (2000). Production, purification and characterization of pectinase from a *Bacillus* sp. DT7. *World Journal of Microbiology and Biotechnology*.

[14] El-Shishtawy R. M., Mohamed S. A., Asiri A. M., Gomaa A.-B. M., Ibrahim I. H., Al-Talhi H. A. (2014). Solid fermentation of wheat bran for hydrolytic enzymes production and saccharification content by a local isolate Bacillus megatherium. *BMC Biotechnology*.

[15] Khairnar Y., B J., Mujapara A. (2009). Study of pectinase production in submerged fermentation using different strains of Aspergillus Niger. *International Journal of Microbiology Research*.

[16] Banu A., Rasheedha M., Devi G. R. K. (2010). Production and characterization of pectinase enzyme from Penicillium chrysogenum. *Indian Journal of Science and Technology*.

[17] Namasivayam E., John Ravindar D., Mariappan K., jiji A., Kumar M., Jayaraj R. L. (2011). Production of extracellular pectinase by bacillus cereus isolated from market solid waste. *Journal of Bioanalysis & Biomedicine*.

[18] Ahlawat S., Mandhan R. P., Dhiman S. S., Kumar R., Sharma J. (2008). Potential application of alkaline pectinase from Bacillus subtilis SS in pulp and paper industry. *Applied Biochemistry and Biotechnology*.

[19] Darah I., Nisha M., Lim S. H. (2013). Enhancement of polygalacturonase production from enterobacter aerogenes NBO2 by submerged fermentation. *Advanced Studies in Biology*.

[20] Kashyap D. R., Soni S. K., Tewari R. (2003). Enhanced production of pectinase by Bacillus sp. DT7 using solid state fermentation. *Bioresource Technology*.

[21] Crotti L. B., Jabor V. A. P., Chellegatti M. A. D. S. C., Fonseca M. J. V., Said S. (1999). Studies of pectic enzymes produced by Talaromyces flavus in submerged and solid substrate cultures. *Journal of Basic Microbiology*.

[22] Beg Q. K., Kapoor M., Tiwari R. P., Hoondal G. S. (2001). Bleach-boosting of eucalyptus kraft pulp using combination of xylanase and pectinase from Streptomyces sp. QG-11-3. *Res. Bullet. Panjab Univer*.

[23] Solis-Pereira S., Favela-Torres E., Viniegra-González G., Gutiérrez-Rojas M. (1993). Effects of different carbon sources on the synthesis of pectinase by Aspergillus niger in submerged and solid state fermentations. *Applied Microbiology and Biotechnology*.

[24] Fawole O. B., Odunfa S. A. (1992). Pectolytic moulds in Nigeria. *Letters in Applied Microbiology*.

[25] Phutela U., Dhuna V., Sandhu S., Chadha B. S. (2005). Pectinase and polygalacturonase production by a thermophilic Aspergillus fumigatus isolated from decomposting orange peels. *Brazilian Journal of Microbiology*.

[26] Rehman H. U., Qader S. A. U., Aman A. (2012). Polygalacturonase: Production of pectin depolymerising enzyme from Bacillus licheniformis KIBGE IB-21. *Carbohydrate Polymers*.

[27] Padhiar J., Das A., Bhattacharya S. (2011). Optimization of process parameters influencing the submerged fermentation of extracellular lipases from Pseudomonas aeruginosa, Candida albicans and Aspergillus flavus. *Pakistan Journal of Biological Sciences*.

[28] Thakur A., Pahwa R., Singh S., Gupta R. (2010). Production, purification, and characterization of polygalacturonase from mucor circinelloides ITCC 6025. *Enzyme Research*.

[29] Sarvamangala R. P., Dayanand A. (2006). Exploration of regional agrowastes for the production of pectinase by Aspergillusniger. *Food Technology and Biotechnology*.

[30] Siddiqui M. A., Pande V., Arif M. (2012). Production, purification, and characterization of polygalacturonase from Rhizomucor pusillus isolated from decomposting orange peels. *Enzyme Research*.

[31] Palaniyappan M., Vijayagopal V., Viswanathan R., Viruthagiri T. (2009). Statistical optimization of substrate, carbon and nitrogen source by response surface methodology for pectinase production using Aspergillus fumigatus MTCC 870 in submerged fermentation. *African Journal of Biotechnology*.

